# Therapeutic Value Assessments of Novel Medicines in the US and Europe, 2018-2019

**DOI:** 10.1001/jamanetworkopen.2022.6479

**Published:** 2022-04-06

**Authors:** Kerstin N. Vokinger, Thomas J. Hwang, Camille E. G. Glaus, Aaron S. Kesselheim

**Affiliations:** 1 Institute of Law, University of Zurich, Zurich, Switzerland; 2 Program on Regulation, Therapeutics, and Law, Department of Medicine, Division of Pharmacoepidemiology and Pharmacoeconomics, Brigham and Women’s Hospital and Harvard Medical School, Boston, Massachusetts

## Abstract

This economic evaluation study assesses the clinical benefit of novel drugs approved in 2018 and 2019 and examines whether drugs approved with special regulatory designations appear to provide meaningful therapeutic value.

## Introduction

The rate of new drug approvals by the US Food and Drug Administration (FDA) has increased over the past decade.^1^ One factor contributing to this increase in number of drugs entering the market is the expedited regulatory review programs that the FDA and European Medicines Agency (EMA) have established to speed access to new treatments.^2^ For example, the accelerated approval (AA) program permits the FDA to approve novel drugs for serious or life-threatening diseases on the basis of changes to surrogate measures that only are reasonably likely to predict clinical benefit.^2^ Surrogate measures are laboratory values that can be observed faster than clinical end points but may not accurately estimate how a patient feels, functions, or survives.^3^ The analogous approval pathway in the European Union (EU) is the conditional marketing authorization (CMA). Special designations in the US and Europe are also available for drugs addressing rare diseases and promote regulatory flexibility when evaluating the evidence.^4^

There has been controversy about the increase in recent drug approvals and therapeutic value of some of these drugs.^4,5^ In this economic evaluation study, we used independent assessments of the clinical benefit of novel drugs approved in 2018 and 2019 and examined whether drugs approved with special regulatory designations appear to provide meaningful therapeutic value.

## Methods

This study adheres to the Consolidated Health Economic Evaluation Reporting Standards (CHEERS) reporting guideline. This study was not submitted for institutional review board approval and informed consent was not sought or required because no patient data were involved, in accordance with 45 CFR §46.

Among all novel drugs FDA-approved in 2018 and 2019—excluding generic, biosimilar, and diagnostic agents—we identified drugs that had also been approved by the EMA by October 2021.^6^ From the FDA and EMA databases, we then extracted therapeutic area (using the World Health Organization’s Anatomic Therapeutic Chemical classification system), date of approval, indication, AA, CMA, and special designation as treating a rare disease.^2^

We used the German (Federal Joint Committee) and French (National Authority for Health) ratings of therapeutic value assessment and defined ratings of moderate or greater added therapeutic value as high and the rest (minor, possible, not quantified, and no or slight benefit) as low.^2^ When multiple ratings were provided for a single drug (eg, for different subgroups), we used the most favorable rating received at the time of the drug’s approval. When health technology assessment agencies reached different outcomes, we used the most favorable rating. We used Excel software version 16.58 (Microsoft) to analyze the data.

## Results

Among the 67 drugs in the final cohort, most were indicated for oncological (26 drugs [39%]), neurological (10 drugs [15%]), or infectious (9 drugs [13%]) diseases. Eight drugs (12%) qualified for AA, and 10 drugs (15%) qualified for CMA. More than one-half (38 drugs [57%]) qualified for rare disease designation in the US, and more than one-third (24 drugs [36%]) did so in the EU (Table).

**Table.** Drugs in the Study Cohort That Were Granted AA or CMA Designation

Active ingredient	Drug name	Approval date		Approval pathway	
		US	EU	US (AA)	EU (CMA)
Burosumab	Crysvita	April 2018	September 2018	No	Yes
Cemiplimab	Libtayo	September 2018	June 2019	No	Yes
Crizanlizumab	Adakveo	November 2019	October 2020	No	Yes
Duvelisib	Copiktra	September 2018	May 2020	Yes	No
Entrectinib	Rozlytrek	August 2019	July 2020	Yes	Yes
Larotrectinib	Vitrakvi	November 2018	September 2019	Yes	Yes
Lorlatinib	Lorbrena	November 2018	June 2019	Yes	Yes
Migalastat	Galafold	August 2019	May 2016	Yes	No
Polatuzumab vedotin	Polivy	June 2019	January 2020	Yes	Yes
Pretomanid	Dovprela	August 2019	July 2020	No	Yes
Trastuzumab deruxtecan	Enhertu	December 2019	January 2021	Yes	Yes
Selinexor	Xpovio	July 2019	March 2021	Yes	Yes

Abbreviations: AA, accelerated approval; CMA, conditional marketing authorization; EU, European Union.

Therapeutic value ratings were available for 57 drugs. Eighteen (32%) had high added therapeutic value, including none of the AA and 1 of the CMA drugs. Ten of 34 drugs (29%) and 10 of 22 drugs (45%) with special rare disease designations from the FDA and EMA, respectively, had high added therapeutic value (Figure).

**Figure.**
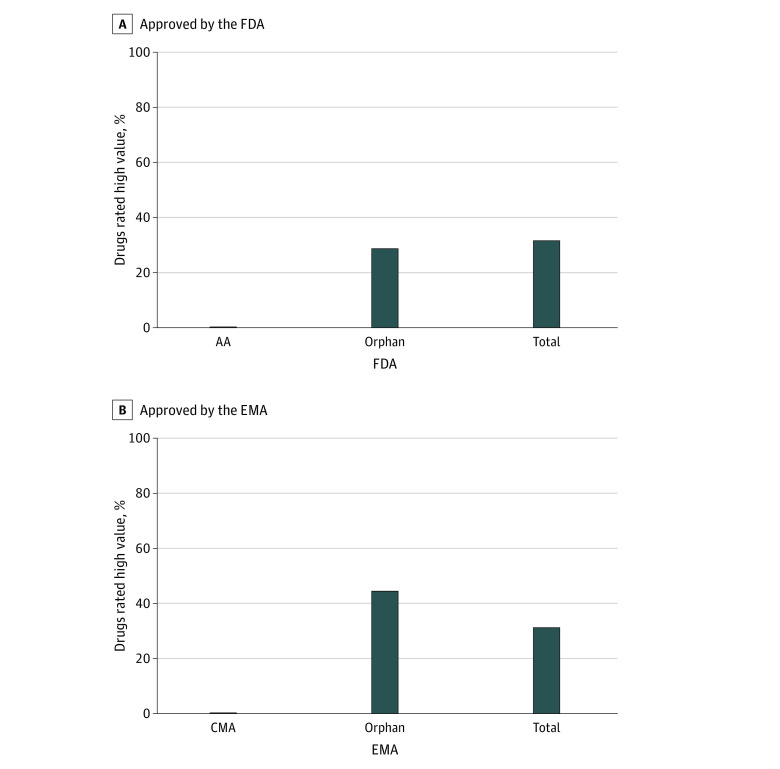
Proportion of New Drugs Approved by the US Food and Drug Administration (FDA) and European Medicines Agency (EMA) Rated as Having a High Therapeutic Value AA indicates accelerated approval; CMA, conditional marketing authorization.

## Discussion

In this economic evaluation study, we found that less than one-third of novel drugs approved by the FDA and EMA in recent years had high added therapeutic value. These results are consistent with a previous study focusing on drugs approved until 2017.^2^ None of the AA or CMA drugs had high added therapeutic benefits. Because we examined only drugs approved in 2018 to 2019, our results may not be generalizable to drugs approved in earlier years. The fact that so few of the expedited drugs or those for rare diseases had a high added therapeutic value supports the need for more scrutiny about which drugs qualify for these programs.
